# Functional-Optical Coherence Tomography: A Non-invasive Approach to Assess the Sympathetic Nervous System and Intrinsic Vascular Regulation

**DOI:** 10.3389/fphys.2019.01146

**Published:** 2019-09-12

**Authors:** Nicholas G. Jendzjowsky, Craig D. Steinback, Robert J. Herman, Willis H. Tsai, Fiona E. Costello, Richard J. A. Wilson

**Affiliations:** ^1^Department of Physiology and Pharmacology, Cumming School of Medicine, University of Calgary, Calgary, AB, Canada; ^2^Program for Pregnancy and Postpartum Health, Physical Activity and Diabetes Laboratory, Faculty of Kinesiology, Sport and Recreation, University of Alberta, Edmonton, AB, Canada; ^3^Department of Medicine, Cumming School of Medicine, University of Calgary, Calgary, AB, Canada; ^4^Department of Community Health Sciences, Cumming School of Medicine, University of Calgary, Calgary, AB, Canada; ^5^Department of Clinical Neuroscience, Cumming School of Medicine, University of Calgary, Calgary, AB, Canada; ^6^Department of Surgery, Cumming School of Medicine, University of Calgary, Calgary, AB, Canada

**Keywords:** sympathetic nervous system, autoregulation, microvascular, vascular, choroid

## Abstract

Sympathetic nervous system dysregulation and vascular impairment in neuronal tissue beds are hallmarks of prominent cardiorespiratory diseases. However, an accurate and convenient method of assessing SNA and local vascular regulation is lacking, hindering routine clinical and research assessments. To address this, we investigated whether spectral domain optical coherence tomography (OCT), that allows investigation of retina and choroid vascular responsiveness, reflects sympathetic activity in order to develop a quick, easy and non-invasive sympathetic index. Here, we compare choroid and retina vascular perfusion density (VPD) acquired with OCT and heart rate variability (HRV) to microneurography. We recruited 6 healthy males (26 ± 3 years) and 5 healthy females (23 ± 1 year) and instrumented them for respiratory parameters, ECG, blood pressure and muscle sympathetic nerve microneurography. Choroid VPD decreases with the cold pressor test, inhaled hypoxia and breath-hold, and increases with hyperoxia and hyperpnea suggesting that sympathetic activity dominates choroid responses. In contrast, retina VPD was unaffected by the cold pressor test, increased with hypoxia and breath hold and decreases with hyperoxia and hyperpnea, suggesting metabolic vascular regulation dominates the retina. With regards to integrated muscle sympathetic nerve activity, HRV had low predictive power whereas choroid VPD was strongly (inversely) correlated with integrated muscle sympathetic nerve activity (*R* = −0.76; *p* < 0.0001). These data suggest that Functional-OCT may provide a novel approach to assess sympathetic activity and intrinsic vascular responsiveness (i.e., autoregulation). Given that sympathetic nervous system activity is the main determinant of autonomic function, sympathetic excitation is associated with severe cardiovascular/cardiorespiratory diseases and autoregulation is critical for brain health, we suggest that the use of our new Functional-OCT technique will be of broad interest to clinicians and researchers.

## Introduction

Abnormal sympathetic nervous system activity damages local vascular regulation, including the ability of the microvasculature to resist associated changes in blood pressure (i.e., myogenic autoregulation) and respond appropriately to local metabolic demands. Concurrently, impaired local microvascular regulation reduces tissue perfusion within vital organs, causing tissue damage and organ dysfunction, triggering sympathetic excitation. Consequently, abnormal sympathetic nervous system activity and impoverished microvascular regulation are associated with, and causal to a host of common cardiovascular, cardiorespiratory and metabolic diseases including sleep apnea, hypertension, metabolic diseases, stroke, heart failure and dementia (Mancia et al., 1999; Barretto et al., 2009; Grassi et al., 2009; Agemy et al., 2015; Gutterman et al., 2016; Doustar et al., 2017; Nelis et al., 2018).

Available methods to measure sympathetic responses suffer from poor time resolution (i.e., norepinephrine spillover), multiple neuronal inputs (i.e., skin temperature, heart rate variability), are invasive (i.e., microneurography) and/or do not completely reflect sympathetic activity (i.e., heart rate variability) (Mancia et al., 1998; Notarius et al., 1999; Jänig and Häbler, 2003; Baumert et al., 2009; Martelli et al., 2014). Methods to measure microvascular responses – especially in relation to cerebral vascular beds – are limited to techniques which suffer from poor cost effectiveness (i.e., MRI), spatial resolution (i.e., trans-cranial doppler) and/or temporal resolution (i.e., MRI/CT), or require some degree of invasiveness (i.e., dye tracer techniques) (Provenzale et al., 2008; Willie et al., 2014; Blair et al., 2016). Our inability to measure these properties efficiently and non-invasively limits early diagnosis, treatment and research of cardiovascular and cardiorespiratory diseases (Mancia et al., 1999; Grassi et al., 2009; Padilla et al., 2014; Gutterman et al., 2016). To solve this issue, we have developed a new approach to measure blood vessel physiology in humans using spectral domain optical coherence tomography (OCT) imaging of ophthalmic vascular beds.

The posterior segment of the eye is an extension of the brain, but with two distinct vascular beds. The vascular beds of the retina and choroid differ in their local vascular responses and sympathetic innervation (Delaey and Van De Voorde, 2000; Nickla and Wallman, 2010). Specifically, blood vessels of the retina appear to be controlled almost exclusively by myogenic and metabolic mechanisms triggered in response to their local environment (Delaey and Van De Voorde, 2000; Nickla and Wallman, 2010); thus, they behave similarly to blood vessels in the brain (Willie et al., 2012, 2014). In contrast, blood vessels of the choroid express an abundance of α-adrenergic receptors and constrict in response to global sympathetic activation (Bill and Sperber, 1990; Delaey and Van De Voorde, 2000; McDougal and Gamlin, 2015). Accordingly, the vascular compartments of the retina and choroid respond differently to conditions which change global sympathetic activity or reflect local metabolic demands (Delaey and Van De Voorde, 2000; Nickla and Wallman, 2010) and therefore reflect differences in vascular regulation.

Modern OCT devices can easily image both the retina and choroid (Fujimoto et al., 2000; Poddar et al., 2014). Optical coherence tomography is a non-invasive, fast, high-resolution and relatively affordable ophthalmic imaging technology used routinely to detect anatomical abnormalities associated with retinopathies in ophthalmic clinics. Near-infrared lasers and sophisticated image reconstruction algorithms allow visualization deep within the tissue with a spatial resolution of ∼4 μm, 3–4 orders of magnitude greater than magnetic resonance imaging (Fujimoto et al., 2000; Poddar et al., 2014). Ophthalmic OCT imaging of the retina has been used to identify late-stage microvascular anatomic markers for several vascular diseases including diabetes and dementia (Agemy et al., 2015; Doustar et al., 2017; Nelis et al., 2018). However, optimization of diagnostic criteria should include microvascular physiology as changes in functional responses likely occur before anatomic abnormalities are detectable. Given the capabilities of OCT to measure the microvasculature and the differential vascular regulation of the retina and choroid, we hypothesized that Functional-OCT imaging of the eye can be used to assess sympathetic and neurovascular reactivity to physiological perturbations.

To test our hypothesis, we used OCT to compare the microvascular responses of the choroid and retina to different conditions (inspiratory challenges and cold pressor test) designed to disentangle sympathetic and local metabolic vascular control mechanisms. The inspiratory challenges were chosen to stimulate the central and peripheral chemoreflexes reflective of reflex stimulation and mimic sympathetic activation encountered with cardiorespiratory diseases; the cold pressor causes sympathetic activation independent of changes to blood gases. Experiments were performed in 11 healthy human participants while recording ventilation, blood pressure, heart rate and direct measures of sympathetic nerve activity using microneurography, the only method to record from the sympathetic nervous system in directly.

## Materials and Methods

### Participants

The study was approved by the Conjoint Health Research Ethics Board at the University of Calgary and University of Alberta and conformed to institutional guidelines and the standards set by the latest revision of the Declaration of Helsinki. Seventeen healthy volunteers (11 male) 18–36 years of age were recruited from the University of Calgary community. All participants were normotensive, non-smokers and free of respiratory, cardiovascular, neurological diseases, and provided written informed consent prior to study. Data from 11 participants (6 male, 5 female) were analyzed; 6 (5 male, 1 female) participants did not complete the protocol because of difficulty locating and maintaining stable sympathetic recordings and/or, the onset of vasovagal reflexes (*n* = 2) associated with microneurography in the seated position as assessed by cardiovascular indices of pre-syncope (Meah et al., 2019).

### Optical Coherence Tomography

Left eyes were scanned with a Cirrus HD 4000 spectral domain optical coherence tomography system (OCT, Zeiss optics, Toronto, ON, Canada). The Cirrus HD 4000 uses near-infrared laser interferometry, enabling an optical reconstruction of a 6 mm^3^ cube of the eye. All OCT scans were acquired by the same operator (RH) with the Cirrus OCT device (software version 6.5.0772). Image acquisition of the Cirrus OCT system, averages 1024 scans within ∼3 s using active eye-tracking features and centred around the fovea, thus standardizing each image to the anatomical characteristics of each individual eye, to produce a single image set. For each participant, the baseline scan was used to establish a slice which encased the large vessels of the choroid as identified by visual inspection. Once anatomical parameters for the slice were established from the baseline scan, the same parameters were maintained for scans from all subsequent perturbations for that subject. The average slice thickness across subjects was 86 ± 3 μm (the inner and outer surfaces of the slice were located 85 ± 6 μm and 171 ± 9 μm, respectively, from the outer portion of the hyper-reflective line corresponding to the retina pigment epithelium). The retina circulation was measured from images obtained from the 3D visualization mode by capturing the aggregate of the retina vessels on a 2D “flattened” image. Retina thickness was measured using the automatic segmentation values of the Cirrus HD OCT software. Two images were collected during each stimulus. A single image chosen from paired scans was selected for each intervention from each participant. Image selection and image analysis were performed independently by different team members (RH, RW, and, NJ, respectively).

### Instrumentation

Peripheral muscle SNA (MSNA) was assessed using microneurography. Briefly, a tungsten recording electrode with an uninsulated 1–5 μm diameter tip was inserted transcutaneously into the peroneal nerve at or distal to the fibular head. A reference electrode was inserted subcutaneously 1–3 cm from the recording site. The recording electrode was manipulated into position within a sympathetic nerve fascicle, which produced pulse-synchronous bursts of activity characteristic of sympathetic vasoconstrictor neurons innervating skeletal muscle. The sympathetic signal was verified by an increase in firing frequency during voluntary apnoea and lack of responsiveness during arousal to a loud noise. The raw sympathetic signal was amplified by a preamplifier (1000×) and secondary isolated amplifier (gain: 10 K; model 662C-3; Iowa University Bioengineering, IA), band-pass–filtered (700–2000 Hz), rectified and integrated (time constant: 0.1 s) as per standardized methods (Hart et al., 2017). All recordings were performed by a trained microneurographer (CS).

Heart rate was measured using a 3-lead ECG. Respiratory flow was monitored by a pneumotachometer (ParvoMedics/Hans Rudolph, UT, United States) and end-tidal gas concentrations were measured via an oxygen and carbon dioxide analyzer (Powerlab, AD Instruments, CO, United States). Blood pressure was measured continuously using finger photoplethysmography (Finometer; Finapres Medical Systems, Amsterdam, Netherlands) and calibrated to baseline blood pressure values obtained using manual sphygmomanometry. Manual blood pressures were assessed at the brachial artery just proximal to the elbow (arm straight or in mild elbow flexion) using a mercury sphygmomanometer and a medium-sized blood pressure cuff performed by a clinician (RH). Calibrated pressure waveforms were analyzed to determine beat-by-beat MAP and systolic and diastolic blood pressures. Blood oxygen saturation was measured with a pulse oximeter (Nellcor N-395 Medtronic, Brampton, ON, Canada).

### Protocol

Participants were instructed to refrain from caffeine (12 h), exercise (24 h), and alcohol (24 h), and ingest their last meal at least 3 h prior to the laboratory visit. All female participants were tested during the midluteal phase (21 ± 2 days) of their menstrual cycle where minimal effects of the menstrual cycle on sympathetic activity have been demonstrated (Usselman et al., 2015); 4/5 were prescribed oral contraceptives.

After arriving at the laboratory, the study protocol was reviewed with the participants, who were then allowed to acclimatize to the environment prior to being instrumented for the study. Once instrumentation was complete, participants rested for 10 min in a seated position during instrumentation and remained seated throughout the entire protocol. Familiarization to the OCT device entailed 2–4 practice scans which were followed by baseline hemodynamic and neurographic recordings for >15 min. Subsequently, respiratory gas challenges of hypoxia (12% O_2__,_ balance N_2_, 3 min), hyperoxia (100% O_2_, 3 min), hyperoxic-hypercapnia (5% CO_2_, balance O_2_, 3 min), hyperpnea (participants were instructed to breathe “hard and fast”-not paced, 3 min), voluntary end-expiratory sub maximal breath hold (∼10 s) and cold pressor test (hand immersed up to the wrist in ∼1°C ice water, 3 min). The hypoxic challenge was always performed last due to the long lasting hemodynamic and sympathetic changes associated with hypoxia (Xie et al., 2001) while all other interventions were administered in random order. Each challenge was interspersed with ≥10 min of rest to allow baseline cardiovascular and neurographic values to return. The cold pressor test was included to augment sympathetic activation without marked blood gas changes to minimize changes in local vascular regulation.

### Data Analysis

Vascular perfusion density (VPD) defined as a static index of perfusion within the image, was calculated for the choroid and retina of each image for each participant within each condition. The resulting images were analyzed offline using Image J software (NIH, Bethesda, MD, United States). Choroid images were obtained from the RPEFIT mode. All images were subject to the same analysis involving the Iterative Self- Organizing Data Analysis Technique Algorithm (ISODATA). This algorithm provides unsupervised determination of a threshold to classify background and objects (Ridler and Calvard, 1978). Images of the retina were obtained from the 3D visualization mode and a 2D flattened image was selected in order to obtain the aggregate vasculature of the retina circulation. All images were processed the same way using a macro written in Image J (RW). The macro filtered out artifacts and used the moment auto-thresholding approach to obtain vessel density values, reflecting retina VPD.

All hemodynamic and neurographic data were stored for offline data analysis (PowerLab/16SP with LabChart 8; AD Instruments, Colorado Springs, CO, United States). Heart rate variability was assessed by utilizing 3-min bins (time of each perturbation) and analyzed with built-in heart rate variability software (LabChart8; AD Instruments, Colorado Springs, CO, United States). Hemodynamic and neurographic responses to gas challenges and cold pressor test were averaged over 2 min (Notay et al., 2016) while the OCT scan occurred during the middle portion of the 2 min segment. Sympathetic activity was analyzed by a custom written semi-automated peak detection software written in VEE (RW) and confirmed by a trained observer (NJ). Burst amplitude and total activity were analyzed after removal of extraneous noise from the recordings based on statistical parameters derived from the signal itself. The software assigns a threshold cut-off for the noise (four standard deviations from the mean) and calculates the area under all spikes above the cut-off; it then derives total neural activity using all spikes within the field of interest. In this way, the amplitude and total activity is normalized within each participant. For all hemodynamic and sympathetic outcomes, relative responses to sympathetic provocations were normalized to baseline. These methods align with established criteria (Hart et al., 2017) and, importantly, remove observer bias.

### Statistics

Differences between sexes were tested with independent *t*-test. Differences between sympathetic provocations were tested with the Friedman’s non-parametric one-way repeated measures ANOVA using raw values (data are presented as percent change from baseline). When significant F-ratios were found, Student Newman Keuls *post hoc* test was used to discern differences from the baseline condition. Relationships between indices were assessed with Pearson product correlation; and select relationships were analyzed with partial correlation in order to account for gender. Multiple regression analysis was performed when more than one index was used to relate to sympathetic nervous activity. Assessment of inter-measurement agreement was completed using Bland-Altman analysis. A *p*-value < 0.05 was considered statistically significant. All data are expressed as mean ± SEM.

## Results

**FIGURE 1:**
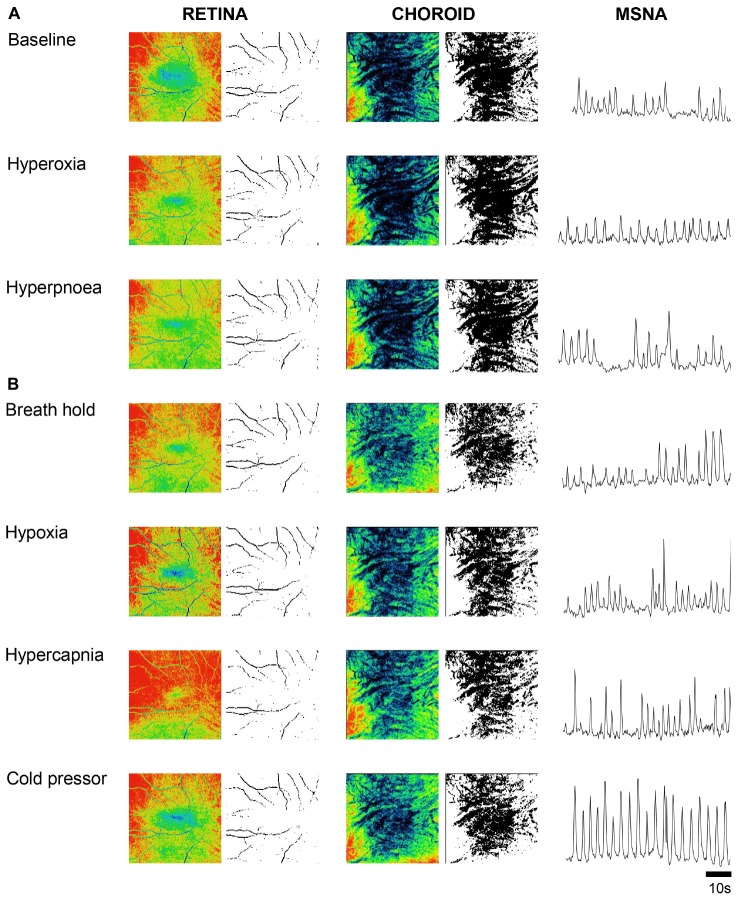
Ophthalmic coherence tomography images demonstrating retina and choroid vascular perfusion and accompanying sympathetic nerve activity from one participant. Raw and processed images from a single participant of the retina (left two columns) and choroid (center two columns) during baseline and various cardiorespiratory challenges. Vascular perfusion is false colored in the raw images; blue and red represents high and low vascular perfusion, respectively. Retina and choroid VPD is demarcated by black pixels in the processed black and white images. Sympathetic activity was recorded from the peroneal nerve using microneurography and is rectified and integrated (right column). When sympathetic activity is low, perfusion in the choroid is high **(A)**; when sympathetic activity is high, perfusion in the choroid is low **(B)**. Note that the retina VPD remains relatively constant in comparison to the choroid.

Figure 1 shows OCT images of the retina and choroid, with associated processed images and muscle sympathetic nerve activity (MSNA), from one participant during baseline and the six conditions designed to disentangle sympathetic from local metabolic and myogenic mechanisms of vascular regulation. Once the participant was appropriately instrumented and seated, each OCT scan took ∼3 s acquiring 1024 scans of 5 μm resolution from a 6 mm^3^ block of tissue. As blood absorbs near-infrared light, blood appears dark in OCT images; to assist in comparisons, the raw images are false colored such that tissues which have high vascular perfusion appear blue whereas tissues with low vasculature perfusion appear red. In the choroid, conditions that result in low sympathetic activity (baseline, hyperoxia and voluntary hyperpnea) tend to increase vascular perfusion (appearing bluer), whereas conditions expected to activate the sympathetic nervous system (voluntary breath hold, hypoxia, hypercapnia and cold pressor test) reduce vascular perfusion (appearing greener). Note that the choroid has far greater vascular perfusion (blue-green) than the retina (yellow-red). Although the effects of the different conditions on the retina are subtler, hypoxia resulted in higher vascular perfusion compared to hyperoxia (during which less perfusion is anticipated). As expected this effect was predominantly demonstrated in distal portions of arteriolar branches (i.e., distal segments dilate or constrict according to the condition).

To use OCT for quantitative assessment of changes in microvascular perfusion, we adopted a measure of vascular perfusion density (VPD) based on pixel intensities in the processed images (Agemy et al., 2015), applied to both the retina and choroid vascular beds. Baseline choroid VPD was identical between sexes; however, baseline retina VPD was greater in females (Table 1). No other sex differences in cardiovascular, ophthalmic or MSNA indices were found within our dataset and, given that burst frequency and total MSNA were similar between males and females during the midluteal phase of the ovarian cycle as previously demonstrated (Usselman et al., 2015), data from males and females were pooled for analysis.

**TABLE 1:** Baseline ophthalmic indices, hemodynamics and sympathetic nerve activity.

**Parameter**	**Male (*n* = 6)**	**Female (*n* = 5)**
Age (years)	26 ± 3	23 ± 1
Height (cm)	177 ± 3	168 ± 6
Mass (kg)	77 ± 4	69 ± 3
HR (beats ⋅ min^–1^)	73 ± 9	72 ± 5
SBP (mmHg)	126 ± 5	120 ± 5
DBP (mmHg)	72 ± 4	69 ± 4
MAP (mmHg)	89 ± 3	82 ± 4
SaO_2_ (%)	98 ± 1	99 ± 1
End-Tidal O_2_ (mmHg)	100 ± 2	102 ± 5
End-Tidal CO_2_ (mmHg)	35 ± 1	33 ± 1
VE (L ⋅ min^–1^)	7.9 ± 0.4	6.5 ± 0.9
RMSSD (ms)	85.70 ± 25.37	47.81 ± 11.26
LF (Hz)	2655.92 ± 1266.57	1503.36 ± 975.34
LF/HF (a.u.)	1.30 ± 0.33	0.44 ± 0.48
SD1 (ms)	55.84 ± 18.33	34.00 ± 7.97
SD2 (ms)	105.28 ± 26.70	43.66 ± 17.26
SNA Burst Frequency (burst ⋅ min^–1^)	20.8 ± 2.9	23.5 ± 3.8
SNA Burst Incidence (burst ⋅ 100 heart beat^–1^)	34.5 ± 4.4	34.4 ± 5.2
SNA (Amplitude, a.u.)	12 ± 2	17 ± 3
SNA (Bursts, a.u.)	108 ± 8	123 ± 17
Retina thickness (μm)	294 ± 7	289 ± 9
Retina VPD (a.u.)	4.0 ± 0.19	5.2 ± 0.45^∗^
Choroid VPD (a.u.)	34.69 ± 3.81	38.08 ± 2.95

*Heart rate variability parameters include: RMSSD, root mean square of successive R–R interval differences (represents parasympathetic mediated changes in heart rate). LF, relative power of the low frequency band of R–R interval Fourier analysis (putative index of sympathetic activity). LF/HF, low frequency to high frequency ratio of Fourier power analysis the R–R interval (putative index of sympathetic: parasympathetic balance). SD1, Poincare plot standard deviation perpendicular line of identity (putative sympathetic index). SD2, Poincare plot standard deviation along the line of identity (putative sympathetic index) (Shaffer and Ginsberg, 2017). ^∗^demonstrates a significant difference between males and females (*p* = 0.013).*

**FIGURE 2:**
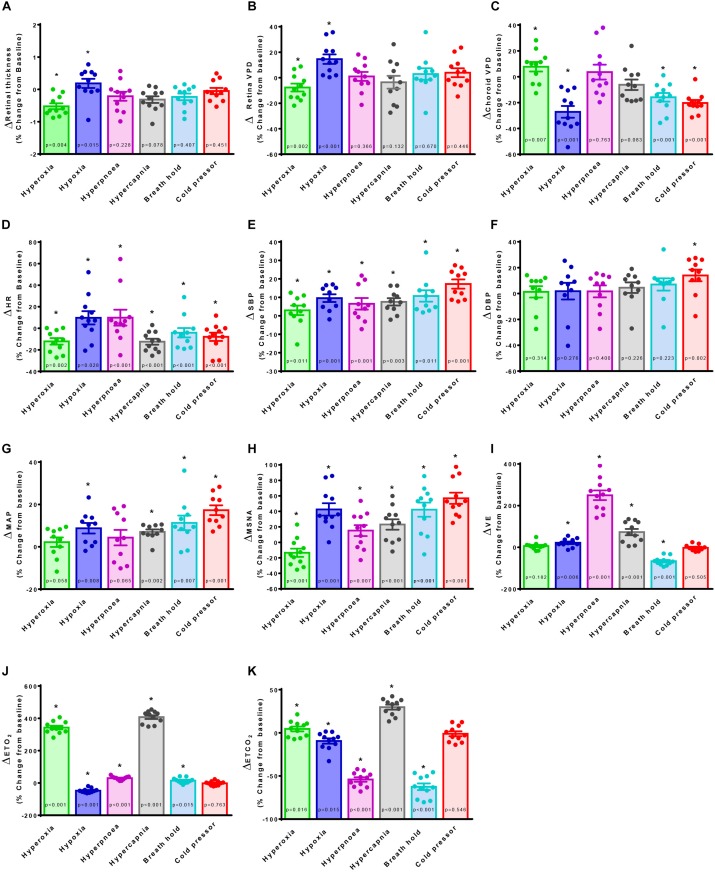
The effect of sympatho-modulation on ophthalmic, cardiorespiratory and sympathetic parameters. Retina thickness **(A)**, retina vascular perfusion density (Retina VPD, **B**), choroid vascular perfusion density (Choroid VPD, **C**), heart rate (HR, **D**), systolic blood pressure (SBP, **E**), diastolic blood pressure (DBP, **F**), mean arterial pressure (MAP, **G**), muscle sympathetic nervous activity (MSNA, **H**), minute ventilation (VE, **I**), end-tidal oxygen content (ETO_2_, **J**), end-tidal carbon dioxide content (ETCO_2_, **K**) in response to hyperoxia (green), hypoxia (blue), hyperpnea (magenta), hypercapnia (gray), breath hold (cyan) or cold pressor test (red). All data are presented as a percentage change from baseline. Absolute data were analyzed with Friedman’s non-parametric repeated measures ANOVA where (^∗^) denotes a significant difference from baseline measures as assessed with Student Newman Keuls *post hoc* test; *p*-values are indicated within each bar.

Retina VPD, choroid VPD, MSNA and cardiorespiratory parameter responses to the six test conditions in 11 participants are illustrated in Figure 2. Hyperpnea increased MSNA mildly (Figure 2H) but had no decisive effect on retina (Figure 2B) or choroid VPD (Figure 2C). The cold pressor test, which caused a substantial increase in MSNA (Figure 2H) without changing blood gases (Figures 2J,K), decreased choroid VPD (Figure 2C). Choroid VPD also decreased with the two respiratory conditions (Figure 2C) which increased MSNA the most (hypoxia and voluntary breath hold). Hypercapnia increased MSNA only mildly (Figure 2H); at best, it caused a slight decrease in choroid VPD (*p* = 0.083, Figure 2C). In contrast, hyperoxia which reduced MSNA (Figure 2H), significantly increased choroid VPD (Figure 2C). Together, these data suggest that choroid VPD is inversely related to SNA: as sympathetic activity increases, vascular perfusion of the choroid decreases and vice versa.

With regards to the retina, hypoxia significantly increased retina VPD compared to baseline (Figure 2B); despite the concurrent increase in sympathetic activation (Figure 2H), blood vessels in the retina vasodilate in response to hypoxia (Eperon et al., 1975; Peeters et al., 1980; Milley et al., 1984; Roff et al., 1999; Ahmed et al., 2001; Wang et al., 2008). Conversely, despite the reduction in sympathetic activity (Figure 2H), hyperoxia reduced retina VPD (Figure 2B) consistent with previous investigations (Eperon et al., 1975; Milley et al., 1984; Nair et al., 2011; Pechauer et al., 2015). Importantly, the cold pressor test which increased sympathetic activity substantially (Figure 2H) without changing blood gases (Figures 2J,K) did not affect retina VPD (Figure 2B). These data confirm that the blood vessels within the retina have dearth sympathetic innervation, are governed primarily by local mechanisms (Nickla and Wallman, 2010; McDougal and Gamlin, 2015) and, suggest that retina vessels reflect cerebral vascular responsiveness (Willie et al., 2012, 2014).

Images were also used to measure the retina thickness; no differences between sexes were identified at baseline (Table 1). Retina thickness changed minimally (<2%) in response to the six conditions; retina thickness was reduced significantly during hyperoxia (consistent with a decrease in retina perfusion volume) and increased with hypoxia (consistent with an increase in retina perfusion volume Figure 2A).

**FIGURE 3:**
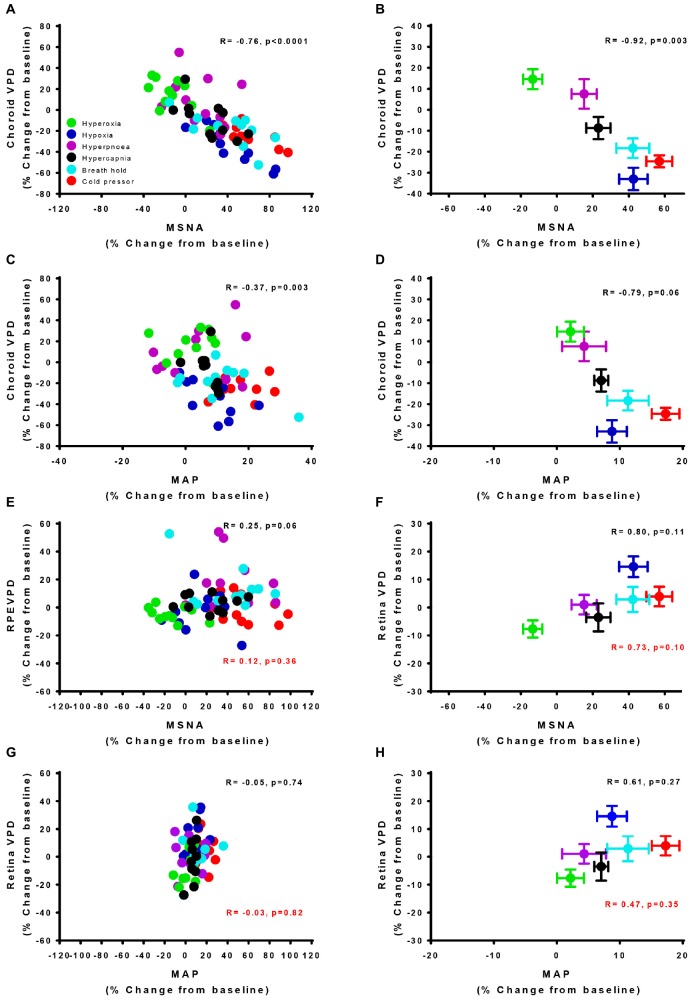
Vascular perfusion density relationships with sympathetic nervous activity and mean arterial pressure for individual and mean data. The relationship of choroid vascular perfusion density (Choroid VPD) to muscle sympathetic nervous activity (MSNA) for individual **(A)** and mean data **(B)** in response to sympathetic provocations. The relationship of Choroid VPD to mean arterial pressure (MAP) for individual **(C)** and mean data **(D)** in response to sympathetic provocations. The relationship of retina vascular perfusion density (Retina VPD) to muscle sympathetic nervous activity (MSNA) for individual **(E)** and mean data **(F)** in response to sympathetic provocations. The relationship of retina VPD to mean arterial pressure (MAP) for individual **(G)** and mean data **(H)** in response to sympathetic provocations. All data are expressed as a percentage change from the initial baseline condition in response to hyperoxia (green), hypoxia (blue), hyperpnea (magenta), hypercapnia (gray), breath hold (cyan), or cold pressor test (red). Pearson R correlation coefficient with accompanying *p*-values are indicated within each panel. For relationships involving the retina, values in red text are inclusive of cold pressor test, values in solid text exclude cold pressor test.

**FIGURE 4:**
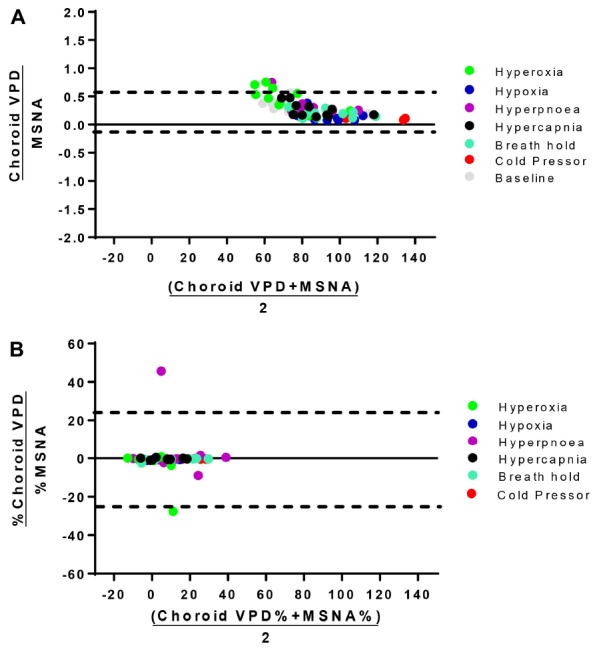
Bland-Altman analysis of choroid VPD versus MSNA. The agreement between absolute choroid vascular perfusion density (Choroid VPD) and absolute muscle sympathetic nerve activity (MSNA, **A**), and percentage change of choroid VPD and percentage change of MSNA **(B)**. Bland Altman analyses are expressed as the ratio of the two measures versus the average of the two measures. Dotted lines represent the lower and upper standard deviation. Absolute choroid VPD and MSNA Bias = 0.27, *SD* = 0.16; Percentage change choroid VPD and MSNA Bias = −4.30, *SD* = 24.48. Hyperoxia (green), hypoxia (blue), hyperpnea (magenta), hypercapnia (gray), breath hold (cyan), cold pressor test (red), baseline (gray).

To further examine the linear correlation between changes in VPD and sympathetic activity, we plotted choroid VPD and retina VPD against MSNA for individual and mean data across all conditions (Figure 3). As shown in Figure 3A with individual data across all conditions, sympathetic activity and choroid VPD had a significant inverse relationship (*R* = −0.76, *p* < 0.0001). Even when accounting for gender with a partial correlation, the relationship between choroid VPD and MSNA remains unchanged (*R* = −0.76, *p* < 0.0001). This relationship was even stronger when mean data for each condition was used (Figure 3B; *R* = −0.92, *p* = 0.003). The predictive capability of choroid VPD is further exemplified by a tight agreement with Bland-Altman analysis for both absolute (Figure 4A) and percentage change values (Figure 4B). For comparison, the relationship between sympathetic activity and MAP in our nominally healthy participants, across all conditions, were *R* = 0.54 (*p* < 0.00001) for all individual and *R* = 0.93 (*p* = 0.007) for mean data. Thus, for individuals, choroid VPD is a better predictor of MSNA than blood pressure. This is further supported by the fact that, though choroid VPD has an inverse relationship with both MSNA and MAP, the relationship between choroid VPD and MSNA is stronger for both individual (Figures 3A vs. 3C) and mean data (Figures 3B vs. 3D). Similarly, within each condition the relationship between choroid VPD and MSNA was stronger than that between choroid VPD and MAP (e.g., hypoxia: *R* = −0.90, *p* = 0.0002 vs. *R* = 0.61, *p* = 0.06; hyperoxia: *R* = −0.65, *p* = 0.03 vs. *R* = 0.23, *p* = 0.53; cold pressor test: *R* = −0.66, *p* = 0.03 vs. *R* = 0.18, *p* = 0.63; voluntary breath hold: *R* = −0.66, *p* = 0.03 vs. *R* = 0.28, *p* = 0.44). Notwithstanding that choroid VPD is a better predictor of MSNA than MAP, combining MAP and choroid VPD responses in a multiple regression analysis improved the power to predict sympathetic responses, compared with using choroid VPD responses alone (individual: *R* = 0.81, *p* < 0.001; mean: *R* = 0.98, *p* < 0.001).

In contrast, the relationships between retina VPD and MSNA or MAP, were weaker when individual data from all six conditions were pooled. This was because across all participants, retina VPD was not responsive to voluntary hyperpnea, hypercapnia, breath hold or the cold pressor test (Figures 3E,G; red stats); controlling for gender with partial correlation, did not alter this relationship (*R* = 0.13, *p* = 0.28). When the cold pressor data were removed (predominant sympathetic stimulus, no metabolic component), a weak positive relationship between retina VPD and MSNA was indicated (Figure 3E, *R* = 0.25, *p* = 0.06 and was not improved with a partial correlation controlling for gender *R* = 0.26, *p* = 0.05); this relationship was improved, but not significantly so, when mean data were used (Figure 3F; excluding cold pressor test, *R* = 0.80, *p* = 0.11). Use of mean data did not reveal a correlation between retina VPD and MAP (Figure 3H). These data suggest that retina VPD is a measure of metabolically driven vascular regulation – primarily vasodilation caused by hypoxia and/or hyperoxia – but not sympathetic activation.

**FIGURE 5:**
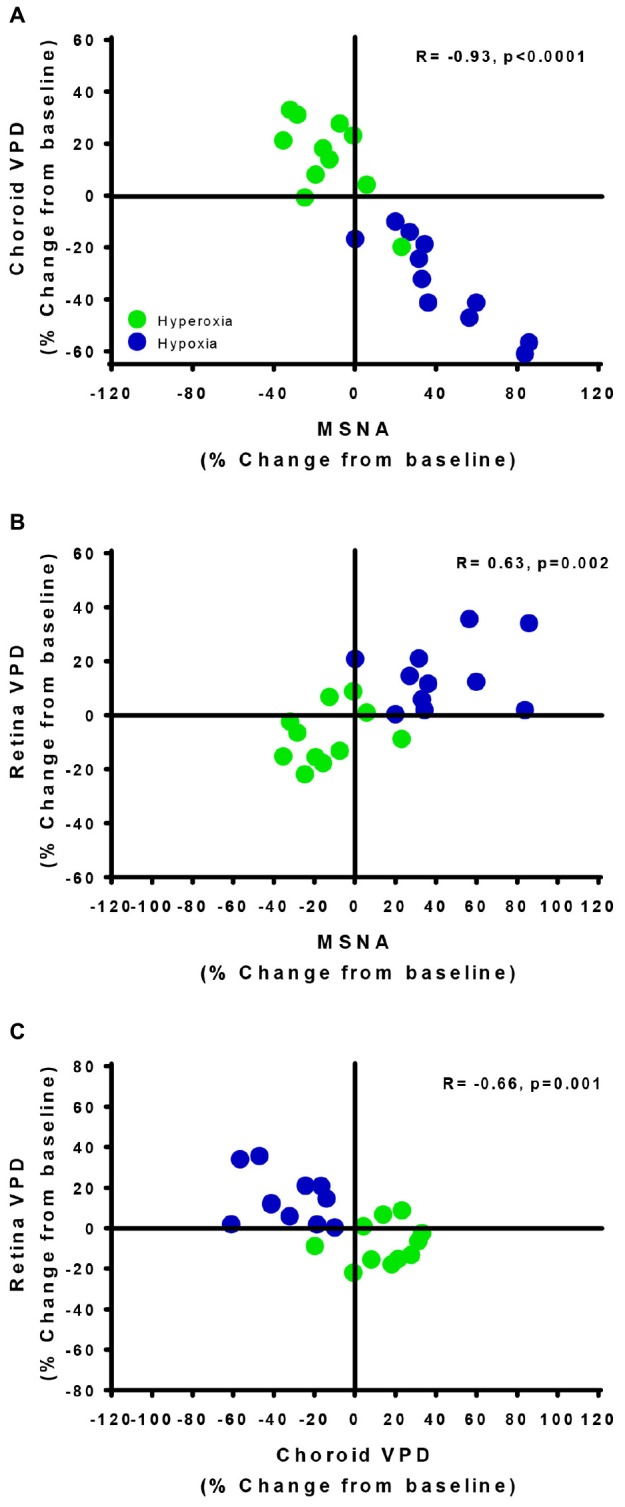
Vascular regulatory mechanisms of autoregulation and sympathetic regulation are revealed by the retina and choroid. The relationships of choroid vascular perfusion density (Choroid VPD, **A**), retina vascular perfusion density (Retina VPD, **B**) to muscle sympathetic nervous activity (MSNA) in response to hypoxia (green) and hyperoxia (blue). The relationships of choroid VPD to retina VPD **(C)**. Each data point represents each participant’s response to hyperoxia (green) and hypoxia (blue) as a percentage change from baseline. Pearson R correlation coefficient with accompanying *p*-values are indicated within each panel.

The divergent vascular regulation between the choroid and the retina in response to changes in inspired oxygen is also demonstrated in Figure 5. Comparing between participants, we found that the greater the increase in MSNA (with hypoxia), the greater the reduction in choroid VPD; and the greater the decrease in MSNA (with hyperoxia), the greater the increase in choroid VPD. Overall, with data from only the hypoxic and hyperoxic conditions, the inverse relationship between MSNA and choroid VPD was extremely strong (Figure 5A, *R* = −0.93, *p* < 0.0001). Generally, retina VPD increased with hypoxia and decreased with hyperoxia, but the correlation to MSNA was lesser (Figure 5B, *R* = 0.63, *p* = 0.002). As expected for two vascular beds differentially controlled by sympathetic inputs and local vascular control mechanisms, the VPD of the choroid and retina were inversely related when interrogated with inspired oxygen (Figure 5C, *R* = −0.66, *p* = 0.001).

Finally, the relationship between choroid VPD and MSNA was far stronger than common indices of HRV widely used as non-invasive, though increasingly controversial (Notarius et al., 1999; Baumert et al., 2009; Goldstein et al., 2011; Heathers, 2014; Martelli et al., 2014), measures of SNA (Individual data- RMSSD: *R* = −0.34, *p* = 0.005; LF: *R* = −0.01, *p* = 0.96; LF/HF: *R* = 0.08, *p* = 0.51; SD1: *R* = −0.34, *p* = 0.005; SD2: *R* = −0.27, *p* = 0.03; Mean data- RMSSD: *R* = −0.25, *p* = 0.63; LF: *R* = −0.37, *p* = 0.48; LF/HF: *R* = 0.15, *p* = 0.77; SD1: *R* = −0.31, *p* = 0.55; SD2: *R* = −0.09, *p* = 0.87).

## Discussion

This study confirms the divergent behavior of the choroid and retina in response to sympathetic stimuli and strongly suggests that ophthalmic Functional-OCT can be used to measure the divergent effects of sympathetic and local regulation of microvascular physiology within neuronal tissue in conscious humans. Our data suggest that the relationship between MSNA and OCT-derived choroid VPD far surpasses that between MSNA and HRV. This illustrates the potential for Functional-OCT as a novel non-invasive method to monitor sympathetic responsiveness in clinical and research settings that are often ill-suited for microneurography and suggests this technique as a new method to assess overall microvascular health.

The data herein where obtained by combining MSNA recording and ophthalmic OCT imaging which raised several technical challenges. MSNA recording and OCT imaging required participants to remain stationary in a seated position while breathing gas mixtures through a mouth piece. The seated position and mouth piece likely increased participant discomfort and compounding the difficulty of making stable MSNA recordings. However, that we found no evidence to suggest that scanning with the OCT device caused additional stress (see “Limitations”). In addition, while requiring MSNA recording, two of our participants experienced vasovagal reflexes that were rapidly resolved by placing in a horizontal position (Meah et al., 2019); this ended the protocol as our participants were required to remain seated. Consequently, we limited our participant pool to *n* = 17, of which we were only able to obtain stable recordings sufficient to complete the protocol in 6 females and 5 males (65%).

The protocol produced alterations in SNA by employing perturbations that primarily stimulate peripheral carotid body chemoreceptor activity (hypoxia), central brainstem chemoreceptor activity with carotid bodies inactivated (hypercapnia with hyperoxic background), combined chemoreceptor activity (voluntary breath hold, i.e., reduced PaO_2_ and increased PaCO_2_; voluntary hyperpnea, i.e., increased PaO_2_ and decreased PaCO_2_), or thermal afferent stimulation (sympathetic stimulation independent of chemoreceptors). MSNA responses to these perturbations were consistent between males and females and with previous human studies measuring MSNA with microneurography during similar sympathetic stressors (Victor et al., 1987; Steinback et al., 2009). Thus, we conclude that our sample size, while limited, is likely sufficient to generalize sympathetic responses to a larger healthy young population.

Across the majority of sympathetic perturbations, changes in choroid VPD of individual participants that completed the protocol were strongly (and inversely) associated with their MSNA responses, consistent with robust choroid vascular sympathetic regulation. This is in accordance with human (Matsuo and Cynader, 1992) and animal (Milley et al., 1984; Riva et al., 1994; Kawarai and Koss, 1998; Steinle et al., 2000; Wikberg-Matsson et al., 2000; Suzuki et al., 2002; Muir and Duong, 2011) investigations demonstrating that the choroid expresses α_1_- and α_2_-adrenergic receptors (Matsuo and Cynader, 1992; Kawarai and Koss, 1998; Steinle et al., 2000; Wikberg-Matsson et al., 2000; Suzuki et al., 2002), and choroid perfusion is reduced with hypoxia (Milley et al., 1984; Muir and Duong, 2011) and increased with hyperoxia (Milley et al., 1984; Riva et al., 1994) in anesthetized animals. Indeed, we found that hyperoxia and hypoxia caused an increase and decrease in choroid VPD, respectively. Moreover, and confirming the utility of the choroid VPD to gauge sympathetic activity, the cold pressor test had little effect on respiratory parameters yet caused a robust MSNA response which was met by an equally robust decrease in choroid VPD. However, the authors note that sympathetic activity is regionalized and further comparisons to other sympathetically innervated tissues may provide further insight into the regulation of global sympathetic activity.

Previous studies suggest that sympathetic outputs to muscle (peroneal), heart and renal vascular beds are differentially regulated (Park et al., 2007; Kuroki et al., 2012; Frithiof et al., 2014). This regulation may be highly variable between different organs and individuals and be a source of disease susceptibility (Simms et al., 2007; Subramanian and Mueller, 2016). Choroid VPD is highly correlated and predictive of MSNA as assessed by linear correlation and Bland Altman analysis. However, unlike microneurography, choroid VPD provides a measure – albeit a surrogate – of sympathetic activity to a neuronal tissue within the central nervous system, not muscle.

With regards to differential regulation between different limbs of the sympathetic nervous system, we note that while overall choroid VPD was inversely proportional to MSNA and strongly correlated, some of our more complex challenges caused disengagement between these variables. For example, hypercapnia on a hyperoxic background (5% CO_2_, 95% O_2_) and voluntary hyperpnea both increased MSNA, yet neither had a significant effect on choroid VPD. Some previous studies also reported no change (Wang et al., 2008; Nair et al., 2011) or an increase (Milley et al., 1984; Geiser et al., 2000; Hétu et al., 2013) in choroid perfusion with increased inspired CO_2_ whereas others reported both increases or decreases (Strang et al., 1974). Previous studies have also reported no difference or a slight decrease in choroid perfusion with hyperpnea/reduced CO_2_ (Strang et al., 1974; Wang et al., 2008). These findings likely reflect the complex systemic effects of these stimuli on central (CO_2_; Wilson and Teppema, 2016), peripheral (O2; Wilson and Teppema, 2016) and metabotropic chemoreceptors (ventilatory work; St Croix et al., 2000), and their differential effects on different limbs of the sympathetic system. In addition to the sympathetic innervation from the superior cervical ganglion, the choroid receives parasympathetic innervation predominantly from the pterygopalatine ganglion (Nilsson, 2000; Steinle et al., 2000; Nickla and Wallman, 2010), but also from the trigeminal ganglion (Nakanome et al., 1995; Nickla and Wallman, 2010) and, the ciliary ganglion (a mixed parasympathetic and sympathetic ganglion; Shih et al., 1999; Nickla and Wallman, 2010; McDougal and Gamlin, 2015). Thus, parasympathetic innervation may have been an additional source of variability in response to the imposed ventilatory stimuli. However, the stimuli employed produced significant increases in MSNA with no change in HRV, some parameters of which are highly suggestive of parasympathetic activity. Therefore, we suggest that the choroid VPD is largely dominated by changes in sympathetic activity.

Comparing choroid VPD and MAP data, we found MSNA responses of individuals to be more strongly associated with changes in choroid VPD than changes in MAP. This suggest that choroid vascular regulation and sympathetic activity is partially uncoupled from systemic arterial pressure. Indeed, partial uncoupling of sympathetic activity from systemic arterial pressure is congruent with one of the primary roles of sympathetic activity in healthy humans: adjusting peripheral vascular resistance to maintain systemic blood pressure, and thus ensuring a constant supply of blood to the brain and other vital organs (Charkoudian et al., 2005).

In contrast to the choroid, retina VPD was not directly correlated with MSNA in individuals, as evidenced by no change in retina VPD with the cold pressor test. That retina VPD and MSNA co-varied in response to some respiratory challenges (hypoxia and hyperoxia) – but not the cold pressor test – suggest that retina vessels are solely governed by local metabolic control mechanisms. For example, during hypoxia the retina blood vessels dilate in response to a metabolic stimulus to increase the supply of oxygen to the surrounding tissue, whereas the converse occurs when the metabolic stimulus is reduced during hyperoxia (Delaey and Van De Voorde, 2000). The retina microvascular responses reported here, mirror cerebrovascular regulation as the large middle cerebral artery reduces blood flow in response to hyperoxia and hypocapnia and increases blood flow to hypoxia and hypercapnia (Willie et al., 2012, 2014). The lack of direct sympathetic regulation in the retina and the poor association in individuals between retina VPD with MAP suggest that Functional-OCT may be used to deliver a quick and relatively low-cost method for assessing the local microcirculatory control mechanisms in a neuronal vascular bed. The response of the retina circulation to metabolic signals is consistent with a recent OCT study in mice demonstrating that retina vessels respond to stimulus−evoked neural activity (Son et al., 2018). As OCT technologies improve so will the accuracy and utility of these measurements.

### OCT – Comparison to Other Technologies

Choroid VPD provides distinct advantages and disadvantages in comparison to other methods of assessing sympathetic activity in humans such as galvanic skin responses, pupil diameter, catecholamine measurements in blood, HRV or microneurography (Mancia et al., 1998; Grassi and Esler, 1999; Jänig and Häbler, 2003; Hart et al., 2017). Distinguishing it from these other techniques and opening new avenues for research, drug discovery and clinical use, choroid VPD provides information about sympathetic control of a vascular bed within the central nervous system. Having the ability to measure from multiple branches of the sympathetic nervous system in humans is likely to be important for research, given animal studies demonstrating differential regulation of different sympathetic outflows (Park et al., 2007; Kuroki et al., 2012; Frithiof et al., 2014; Subramanian and Mueller, 2016). Moreover, as regulation of cerebral blood flow plays a critical role in chronic diseases such as sleep apnea, hypertension and cognitive decline (Willie et al., 2014), having a method to measure sympathetic (and local microvascular) control mechanisms of cerebral vascular bed is likely to provide important indices of cerebral microvascular function (Willie et al., 2012, 2014). Another distinct advantage of OCT for sympathetic assessment is that the retina, lacking sympathetic innervation, can provide a convenient internal control.

On a practical level, unlike catecholamine measurements in blood, OCT is non-invasive and provides a temporal resolution better than or equal to fast blood sampling (∼3 s), but OCT does not allow continuous sampling like microneurography and HRV. Although microneurography provides a direct measurement of SNA and is the only method to directly record sympathetic activity directly and in real-time in humans, its invasive nature and other operational limitations (e.g., inability to determine electrode proximity to nerve fascicle and therefore calculate accurate burst amplitude, and difficulty obtaining a stable signal) minimize its use in research and clinical settings. Galvanic skin responses and pupil diameter appear to be associated with autonomic activity but may suffer from sensory, emotional and higher cortical influences (Bradley et al., 2008; Smith and Johnson, 2016). Heart rate variability, another non-invasive technique available for inferring sympathetic activity is limited by the inherent challenge of parsing out sympathetic from competing parasympathetic signals, effects of respiration and, intrinsic regulatory mechanisms that contribute to heart rate. This diminishes the accuracy of HRV as a simple index of sympathetic activity (Notarius et al., 1999; Baumert et al., 2009; Goldstein et al., 2011; Heathers, 2014; Martelli et al., 2014), as is exemplified in our comparison between measures of HRV and MSNA. In summary, the use of multiple measurements of sympathetic activity is necessary as no single method is likely to reveal complete information regarding the status of autonomic activity in humans.

Current methods to image/assess microcirculatory function are insufficient to resolve microvascular morphometry and, MRI and CT machines are too costly to use broadly (Neeman and Dafni, 2003). Other methods which rely on near-infrared light hemo-absorption do not accurately reflect differences between vascular branches, and the amount and depth of tissue penetration is unknown, compromising repeatability. The image depth and resolution of OCT, as well as the ability to differentiate sympathetic (choroid) and local/metabolic vascular control (retina) mechanisms, allow for a largely independent microcirculatory assessment.

### Limitations

The use of the Functional-OCT provides many advantages over other techniques as listed above. However, the technique has several potential limitations which require consideration. (1) This technique requires the head to be fixed in relation to the OCT machine. Modern OCT devices include eye tracking software that allow movement of the rest of the body, which minimize such distortions. (2) Although OCT is used clinically, the scan itself may impose stress. To gain a bearing on this possibility, we compared our MSNA data (a measure of stress response) before and during OCT scans whilst breathing room air (normal baseline condition). The change in MSNA between the scan vs. no scan condition was −2.7 ± 4.6% and not different between conditions (analyzed with paired two-sided *t*-test, *p* = 0.34). (3) Another potential issue is that shadowing from large diameter vessels in the retina may obscure the signal used to measure choroid responses. In the current study, we circumvented this issue by assessing each choroid image for shadow of retinal vessels. In the few cases where this was a factor, we excluded the regions affected from the choroid analysis. (4) Changes to perfusion in one vascular bed may affect the perfusion in the other vascular bed. Sympathetic activation of the choroid does not affect retina VPD, as the cold pressor test had an independent effect on the choroid. We cannot exclude the converse possibility that changes in retina VPD affect choroid VPD. Nonetheless, we note that responses of the choroid and retina to hypoxia and hyperoxia are diametrically opposed, thus any effect on the choroid of microcirculatory changes in the retina in response to metabolic stimuli must be limited to magnitude.

## Conclusion

The data herein demonstrate the ability of OCT to assess the divergent behavior of ophthalmic vascular beds to provocations designed to disentangle sympathetic from local vascular control mechanisms. Thus, Functional-OCT may provide a novel, fast, non-invasive and relatively affordable imaging solution to assess sympathetic activity and microvascular function, supplementing currently available autonomic measurements. Given dysregulation of the sympathetic nervous system and metabolic/myogenic vascular control are linked to, and often precede clinical identification of serious cardiorespiratory disease (Mancia et al., 1999; Triantafyllou et al., 2015), we suggest Functional-OCT will be of broad interest to clinicians and researchers alike.

## Data Availability

The datasets generated for this study are available on request to the corresponding author.

## Ethics Statement

The studies involving human participants were reviewed and approved by the University of Calgary Conjoint Health Research Ethics Board. The patients/participants provided their written informed consent to participate in this study.

## Author Contributions

NJ, RH, WT, FC, and RW were responsible for research conception and edited and approved the final version of the manuscript. NJ, RH, CS, and RW designed and performed the experiments. NJ, RH, and RW analyzed the data and interpreted the results of the experiments. NJ and RW prepared the figures and drafted the manuscript.

## Conflict of Interest Statement

NJ, CS, RH, WT, FC, and RW have submitted a patent application for the use of optical coherence tomography as a method to interrogate cardiorespiratory function.
